# Performance analysis of multi-angle QAOA for $p > 1$

**DOI:** 10.1038/s41598-024-69643-6

**Published:** 2024-08-14

**Authors:** Igor Gaidai, Rebekah Herrman

**Affiliations:** Department of Industrial and Systems Engineering, University of Tennessee at Knoxville, 37996 Knoxville, TN USA

**Keywords:** Information theory and computation, Computer science

## Abstract

In this paper we consider the scalability of multi-angle QAOA with respect to the number of QAOA layers. We found that MA-QAOA is able to significantly reduce the depth of QAOA circuits, by a factor of up to 4 for the considered data sets. Moreover, MA-QAOA is less sensitive to system size, therefore we predict that this factor will be even larger for big graphs. However, MA-QAOA was found to be not optimal for minimization of the total QPU time. Different optimization initialization strategies are considered and compared for both QAOA and MA-QAOA. Among them, a new initialization strategy is suggested for MA-QAOA that is able to consistently and significantly outperform random initialization used in the previous studies.

## Introduction

The Quantum Approximate Optimization Algorithm (QAOA)^1–4^ is a promising variational quantum algorithm designed to find approximate solutions to combinatorial optimization problems. With the appropriate transformations^5,6^, the optimized cost function can be translated to the cost Hamiltonian *C*, and the original problem can be reformulated as finding the maximum eigenpair of *C*.

The basic version of QAOA finds large eigenpairs of *C* as follows. First, a second Hamiltonian *B*, called the mixing Hamiltonian, is introduced. It is usually defined as a sum of Pauli *X* operators applied to each qubit,

1 $$\begin{aligned} B = \sum _{i} X_i, \end{aligned}$$

although other mixers can be found in the literature^7–10^. Then an equal superposition state $\left| + \right\rangle ^{\otimes n}$ (an eigenstate of *B*) is subjected to alternating evolution under the cost (*C*) and mixing (*B*) Hamiltonians for a total of *p* layers.

2 $$\begin{aligned} \left| \gamma ,\beta \right\rangle = U\!\left( B, \beta _p \right) U\!\left( C, \gamma _p \right) ... U\!\left( B, \beta _1 \right) U\!\left( C, \gamma _1 \right) \left| + \right\rangle ^{\otimes n}, \end{aligned}$$

3 $$\begin{aligned} U\!\left( B, \beta \right) = e^{-i \beta B}, \end{aligned}$$

4 $$\begin{aligned} U\!\left( C, \gamma \right) = e^{-i \gamma C}. \end{aligned}$$

The total number of layers (*p*) is a convergence parameter of the algorithm and the individual evolution times of each Hamiltonian ($\gamma _1... \gamma _p$ $\beta _1... \beta _p$, also known as QAOA angles) are selected to maximize the expectation of the cost Hamiltonian in the final QAOA state $\left\langle \gamma , \beta \right| C \left| \gamma , \beta \right\rangle$, usually via a classical optimizer. Finally, a series of repeated measurements of $\left| \gamma ,\beta \right\rangle$ in the computational basis yields a bit string with a cost of at least $\left\langle \gamma , \beta \right| C \left| \gamma , \beta \right\rangle$.

QAOA has been a subject of numerous recent studies (e.g.^11–23^), but many challenges still exist. One of such challenges is an efficient implementation of QAOA on the existing Noisy Intermediate Scale Quantum (NISQ) devices. NISQ devices suffer from high levels of noise^24,25^, which means that only circuits of limited depths^26^ can be executed before the qubits lose coherence and the result is completely dominated by noise. The number of QAOA layers necessary for efficient solution of practically relevant problems significantly exceeds the capacity of the modern quantum hardware. Recent work has studied methods for decomposing practical-sized problems to fit on hardware with fewer qubits, however some of these methods have drawbacks such as trying to piece together disjoint subproblem solutions^27–29^. Therefore, to make QAOA more useful on NISQ devices it is important to design a version of QAOA that minimizes the necessary number of layers.

One way to achieve this is to allow independent evolution times for each term of the cost and mixer Hamiltonians, which changes the definitions of Eqs. (3) and (4) to

5 $$\begin{aligned} U\!\left( B, \vec {\beta } \right) = e^{-i \sum _i \beta _i X_i} = \prod _i e^{-i \beta _i X_i}, \end{aligned}$$

6 $$\begin{aligned} U\!\left( C, \vec {\gamma } \right) = e^{-i \sum _i \gamma _i C_i} = \prod _i e^{-i \gamma _i C_i}. \end{aligned}$$

Obviously, the performance of this modification cannot be worse than that of the original QAOA, since any QAOA result can be reproduced simply by setting all $\gamma _i = \gamma$ and all $\beta _i = \beta$, but how much better can it be? Does it justify the cost of having to optimize a significantly larger number of parameters?

This idea was originally suggested by Farhi et al.^30^ who demonstrated optimistic, but very limited results. Later on, the performance of this modification, dubbed Multi-Angle QAOA (MA-QAOA), was further investigated on a larger data set and analyzed in Refs.^31,32^, but the analysis was limited to $p = 1$. A similar recent development^33^ suggested to combine the ideas of MA-QAOA with an XY-mixer^8^ to achieve even better performance, but they also considered the case of $p = 1$ only. Other related works include additional parameters on well-defined subproblems^34^ and adding problem-independent ansatz layers with multiple parameters^35^.

In practice we would like to take advantage of as many layers as our hardware can support to achieve the desired performance level, which can extend beyond $p = 1$ even on NISQ devices. How many layers will we need for that and how well does MA-QAOA scale beyond $p = 1$? Can we realistically find good angles even as *p* gets large? All of these questions need to be addressed to establish if MA-QAOA is useful for practical implementations on NISQ devices and the goal of this paper is to provide the answers to them.

## Results

The performance of the methods considered in this paper is compared based on the approximation ratio that they can achieve for a given instance of the unweighted MaxCut problem, which is to partition the vertices of a graph into two subsets such that the number of edges between the sets is maximized. MaxCut is an important problem with a multitude of real-world applications (e.g., circuit design, statistical physics, image processing)^36,37^

The approximation ratio is defined in the usual way as the ratio of the largest found expectation of the cost Hamiltonian to the maximum achievable cut on a given graph (found by brute-forcing through all possible partitions):

7 $$\begin{aligned} AR = \frac{\left\langle \gamma , \beta \right| C \left| \gamma , \beta \right\rangle }{C_{max}}. \end{aligned}$$

The approximation ratio is a number in the range of [0, 1].

The graphs for the MaxCut problem were generated randomly with fixed number of nodes and edge probability (Erdös–Rényi model). In order to explore how the performance of the methods scales with respect to the graph characteristics we generated a total of 7 data sets with 1000 connected, non-isomorphic graphs of specific *QAOA covering depth* in each (also referred to as *c-depth*, for brevity), which is defined as a minimum number of QAOA layers necessary for it to see the whole graph (starting from any edge)^1^. This terminology is derived from Ref.^38^, where the authors discuss the minimum number of iterations *p* that QAOA needs to “cover” the entire graph. As such, it is expected to correlate with the performance of QAOA. More specifically, c-depth is defined as the maximum depth of edge-BFS (i.e., Breadth First Search through the edges instead of nodes), taken over all edges.

This metric is closely related to the more conventional graph diameter as

8 $$\begin{aligned} \text{diameter} - 1 \le \mathrm {c{\text{-}}depth} \le \text{diameter} + 1, \end{aligned}$$

where the graph diameter is the maximum shortest distance between all pairs of vertices in the graph. Therefore, graph diameter and c-depth can be regarded as approximately equal. The exact diameter distribution and other characteristics of our data sets are summarized in Table 1.

**Table 1:** Characteristics of the seven data sets considered in this paper.

Set #	Nodes	Edge probability	c-depth	#(diameter = c-depth − 1)	#(diameter = c-depth)	#(diameter = c-depth + 1)
1	9	0.6	3	663	332	5
2	10	0.6	3	762	237	1
3	11	0.6	3	799	201	0
4	12	0.6	3	823	176	1
5	12	0.2	4	32	481	487
6	12	0.1	5	7	223	770
7	12	0.1	6	12	211	777

The last three columns show the number of graphs with specific diameter within each data set.

The results of the quantum algorithms compared in this work are calculated on a classical state vector simulator implemented in Python. The source code of the simulator and the graph files are available at Ref.^39^.

### Selection of angles for QAOA

In general, it is difficult to find optimal QAOA parameters for the MaxCut problem analytically because the expected value function is a complex trigonometric function that depends on the degree of each vertex in the graph^40^. Therefore, the optimal parameters are typically found by numerical optimization procedures.

One of the biggest challenges with this approach is the problem of selecting good initial angles for such optimization procedures. Selecting random initial angles may require an exponential (in *p*) number of restarts to find the best angles due to a large number of local minima and barren plateaus in the optimization landscape of QAOA, especially for large problem sizes and at high depth^41,42^. As such, a large number of publications in the field has been devoted to finding heuristic strategies for selection of good initial angles^43–50^.

For the sake of fair comparison with MA-QAOA, we wanted to use the best angles that we can find for QAOA within a reasonable budget of optimization attempts. Therefore, in this section we will compare empirical performance of several such heuristics reported in the literature and select the one that performs best. Specifically, the best heuristic is defined as the one that is able to exceed $\text{AR} = 16 / 17 \approx 0.941$ for all graphs in a given data set, using the smallest number of QAOA layers. The number 16/17 was chosen because it is NP-hard to approximate MaxCut with $\text{AR} > 16 / 17$^33,51,52^, therefore this level, in general, cannot be achieved with polynomial amount of resources (time) classically and it is interesting to see how much resources (number of layers) QAOA needs for the same task.

**Figure 1:**
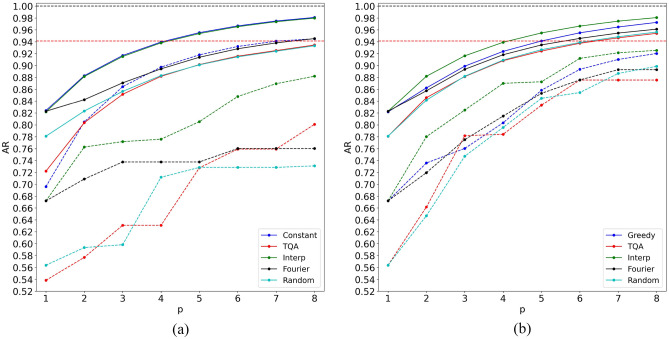
Comparison between different initialization heuristics for QAOA. Solid (dashed) lines show average (worst case) approximation ratio. The horizontal red dashed line shows the desired AR = 16/17.

The heuristics are compared on the data set with 9 nodes (the first row of Table 1). Each method was used to calculate all values of *p* consecutively, until one of them exceeds $\text{AR} = 16 / 17$ on every single graph in every data set. If a method failed to find better angles at a given level *p*, then the best angles found on level $p - 1$ are appended with zeros, and are declared as the best at level *p*. Therefore, the performance of each method can only increase monotonically with *p*. The average and worst case performances of the considered methods are shown in Fig. 1. The left frame shows the case when only 1 optimization attempt was allowed for each value of *p*. On the right frame, the methods were allowed to perform *p* optimizations at a given value of *p*.

The following heuristics were considered: Constant^17^, TQA^46^, Interp^43^, Fourier^43^, Greedy^48^, Random.

The first considered heuristic, labeled as “Constant” here, was taken from Ref.^17^. There, the authors suggest to initialize all values of $\gamma$ with − 0.01 and all values of $\beta$ with 0.01, i.e. a fixed constant that does not depend on anything and has opposite signs on $\gamma$ and $\beta$. We examined convergence with constants in the set $\{0.01, 0.05, 0.1, 0.2, 0.4, 1\}$ (see Supplemental Information for details) and found that QAOA converged fastest when the value was set to 0.2 and appeared to decrease with the distance away from 0.2. This constant was used to generate the corresponding line in Fig. 1. This heuristic is only plotted in the left frame of Fig. 1, since it cannot make use of more than 1 optimization attempt.

The next heuristic, labeled as “TQA” (Trotterized Quantum Annealing), Ref.^46^ assumes, as the name suggests, that good starting angles can be obtained by using linear parameter schedules typical for quantum annealing. Specifically, the values of $\gamma$ are given by $\gamma _i = (i - 0.5)\Delta t / p$, and the values of $\beta$ are given by $\beta = \Delta t - \gamma$, where $\Delta t$ is the only unknown parameter, found by optimization in the one-dimensional $\Delta t$-space, after which the angles corresponding to the optimal $\Delta t$ are used as a starting point for the full 2*p*-dimensional QAOA optimization. In the case of *p* optimization attempts (the right frame of Fig. 1), the first one-dimensional optimization was not counted in the optimization budget as a separate optimization. Additionally, since the number of local minima in the one-dimensional space can be rather limited, if the optimization procedure returned the value of $\Delta t$ that has already been tried, then a random new value of $\Delta t$ was selected. In our experience, we observed that starting the full optimization from a random non-optimal value of $\Delta t$ can often perform better than starting from the optimal one.

The next two heuristics are taken from Ref.^43^ and labeled as “Interp” and “Fourier”. These heuristics are similar in that they assume that the optimal angles change smoothly and re-evaluate the best angles found at level $p - 1$ on a denser grid or use Fourier transform to generate a good starting guess for level *p*. The extra optimization attempts on right frame of Fig. 1 are used to apply random perturbations to the best angles found at level $p - 1$ and start the optimization from there, in accordance with the prescription of Ref.^43^.

Another heuristic, labeled “Greedy” can be found on the right frame of Fig. 1 only, and replaces “Constant” from the left frame. This heuristic (introduced in Ref.^48^) is based on the idea that one can always insert an “empty” QAOA layer (i.e. a layer with $\gamma _i = \beta _i = 0$) before or after any other QAOA layer, which will have no effect on the expectation value of the cost Hamiltonian. As such, these zero insertions to the angle vectors can be used to generate good initial guesses for level *p*, using the best angles found at level $p-1$. Indeed, starting the optimization from such angles guarantees that the expectation value achieved at level *p* will be no worse than the one achieved at level $p-1$. There are *p* possible choices for the insertion position to angles found at level $p-1$, therefore *p* optimizations are necessary to explore all initial guesses obtained in this way, which, in fact, determined the choice of the optimization budget for the right frame of Fig. 1.

Finally, the last considered method is to select the angles randomly, which defines the lowest reasonable performance threshold for any method. This method was used as an experimental control against which to compare the other initialization heuristics.

For all heuristics, except Greedy, L-BFGS-B optimization method from scipy.optimize.minimize python package was used. For Greedy, we used Nelder-Mead, since BFGS is a first-order method and is not suitable for optimization from a transition state, where the first-order derivatives are zero. For heuristics that require the best angles from the previous layer (Interp, Fourier and Greedy), at $p = 1$, we used a random initialization with 10 attempts (in both frames of Fig. 1) to start them with globally optimal angles.

As one can see from Fig. 1, Constant demonstrates superior average and worst-case performance for all *p*, even compared to methods that were allowed *p* optimization attempts. Interp has nearly the same performance on average, but is less consistent and does not do as well in the worst case even with added perturbations (on the right frame). Constant was the first to reach the NP-hard region of $\text{AR} > 16/17$ in the worst case at $p = 8$ and thus, Constant was selected to represent the performance of QAOA in the comparison against MA-QAOA (“Performance comparison between QAOA and MA-QAOA” section).

### Selection of angles for MA-QAOA

In the previous MA-QAOA studies^30–32^ the initial angles were selected randomly. However, as seen in the “Selection of angles for QAOA” section, other techniques for finding initial angles tend to significantly outperform random initialization. Is it possible to adapt some of the QAOA heuristics to MA-QAOA? Will they still work well? In this section we introduce several new angle strategies for MA-QAOA and answer these questions.

The primary candidates for adaptation are the ones that perform best for QAOA. Looking at Fig. 1, we select Constant and Interp as such. Constant applies to MA-QAOA straightforwardly (set all $\gamma _i = 0.2$, and all $\beta _i = -0.2$). For Interp, we interpolate each angle $\gamma _i$ and $\beta _i$ separately, in the same way as it was for QAOA.

Another thing that we could try for MA-QAOA is to start the optimization from the optimal angles found by QAOA (with the Constant strategy, as discussed in the previous section), which we will call “QAOA Relax”.

To determine the role of quality of angles in QAOA Relax strategy, we also consider “Random QAOA”, where the initial values of $\gamma _i$ and $\beta _i$ are random, but equal within the same layer, as in QAOA. Finally, the last strategy is a completely random independent guess, which was the approach used in the previous studies^30–32^.

**Figure 2:**
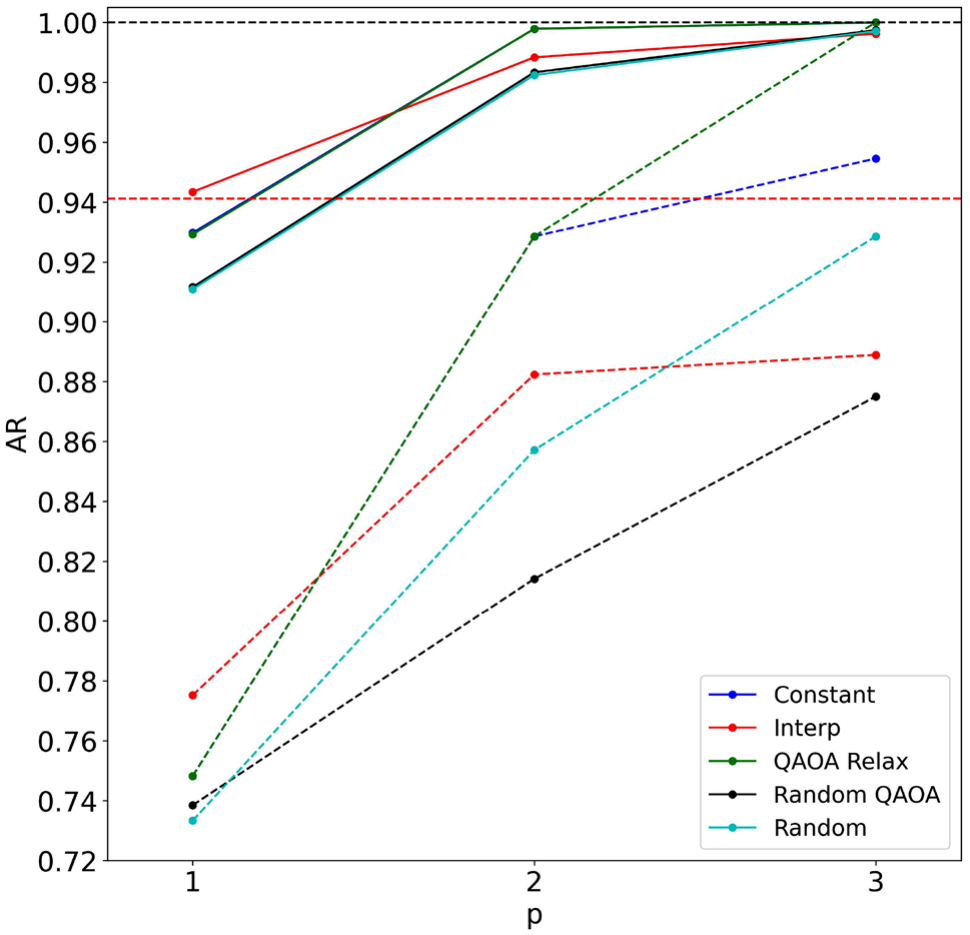
Comparison between different initialization heuristics for MA-QAOA on the data set with 9 nodes. Solid (dashed) lines show average (worst case) approximation ratio. The horizontal red dashed line shows the desired AR = 16/17.

The results of these strategies are shown in Fig. 2 on the nine-vertex data set. Random QAOA and Random strategies show nearly the same performance on average, both worse than QAOA Relax. This indicates that simply keeping the angles the same within each layer does not improve performance and that the quality of QAOA angles for the QAOA Relax strategy is important to achieve quick convergence.

In contrast to QAOA, Interp does not perform well as an MA-QAOA initialization approach. In fact, the AR with the Interp initialization strategy gets even worse than the Random initialization strategy by $p = 3$, which indicates that optimal MA-QAOA angles tend to not change smoothly.

Both Constant and QAOA Relax are nearly identical on average and both reach the target convergence at $p = 3$, but QAOA Relax performs better in the worst case and is able to solve all MaxCut problems in the data set exactly. As such, we select it as the best strategy for comparison with QAOA below.

### Performance comparison between QAOA and MA-QAOA

**Figure 3:**
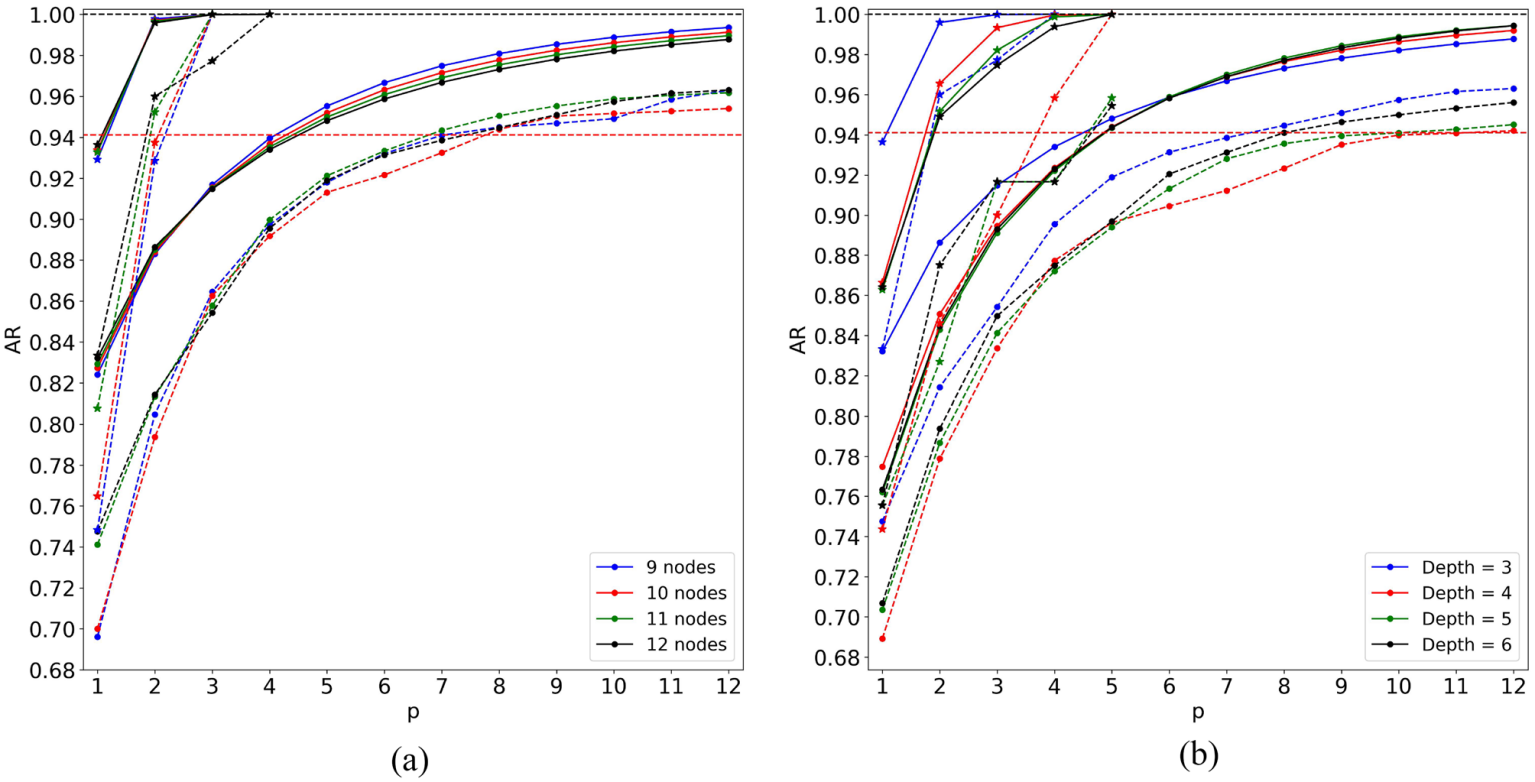
Approximation ratio as a function of *p* obtained with the best angle selection strategies for QAOA and MA-QAOA. Solid (dashed) lines show average (worst case) approximation ratio. Circles (stars) show the results of QAOA (MA-QAOA). The horizontal red dashed line shows the desired AR = 16/17.

Using the best angle strategies picked in the two previous sections (Constant for QAOA and QAOA Relax for MA-QAOA), we calculated the AR on all data sets from Table 1. The results of this are shown in Fig. 3. As one can see, the average AR of both QAOA and MA-QAOA converges to 1 smoothly. The worst case AR is generally less smooth, but still follows a similar trend. At sufficiently large values of *p*, the performance of both QAOA and MA-QAOA expectedly drops as the system size is increasing, but MA-QAOA is less affected by it. Note, that at low values of *p*, the order of the lines in Fig. 3a is reversed, which indicates that the performance of QAOA-like methods in general cannot be adequately judged based on the results for $p = 1$ only, and convergence with respect to *p* has to be analyzed.

Interestingly, from Fig. 3b, one can see that QAOA converges faster for graphs of larger c-depth, while for MA-QAOA the behavior is the opposite. This might prompt the conclusion that QAOA could become better than MA-QAOA for graphs of large c-depth. Note, however, that the maximum c-depth is limited by the number of nodes in a graph and increasing the number of nodes has the opposite and stronger effect on the performance of QAOA. Additionally, the performance of MA-QAOA cannot be worse than performance of QAOA since we start the optimization from the optimal QAOA angles.

In both frames of Fig. 3, we see that MA-QAOA achieves substantially larger ARs and thus converges much faster than QAOA. In particular, the number of layers necessary to converge the average AR required by QAOA was 5 for all data sets, while MA-QAOA was able to achieve the same performance level in 2 layers, reducing the depth of the circuit by a factor of 2.5. The number of layers necessary for the worst case AR is more variable and is given in Table 2. As one can see, in this case MA-QAOA allows to reduce the depth of the circuit by a factor of up to 4. As a consequence of the trends described above, the depth reduction factors tend to increase with the graph size (sets 1–4), but decrease with c-depth (sets 4–7).

**Table 2:** The number of layers necessary to converge the worst-case approximation ratios for QAOA and MA-QAOA.

Set #	# layers QAOA	# layers MA-QAOA	Depth reduction factor
1	8	3	2.7
2	8	3	2.7
3	7	2	3.5
4	8	2	4.0
5	12	4	3.0
6	11	5	2.2
7	9	5	1.8

The set numbers are the same as in Table 1.

### Prospects of MA-QAOA in fault-tolerant era

In the previous section we demonstrated that the depth of QAOA circuit can be made several times smaller by using MA-QAOA. But what if we have a hypothetical fault-tolerant computer and the depth does not matter as much anymore? In that case the new efficiency metric that one can define instead of circuit depth, is the total time it takes to solve the problem, which can be approximated as the number of calls to QPU times depth of the corresponding circuits. Both QAOA and MA-QAOA have the same depth per layer, therefore for the sake of comparison between them, one can measure circuit depth simply in the number of QAOA layers.

9 $$\begin{aligned} \text{Cost} = n_c \times p, \end{aligned}$$

where $n_c$ symbolizes the number of calls to a QPU.

**Figure 4:**
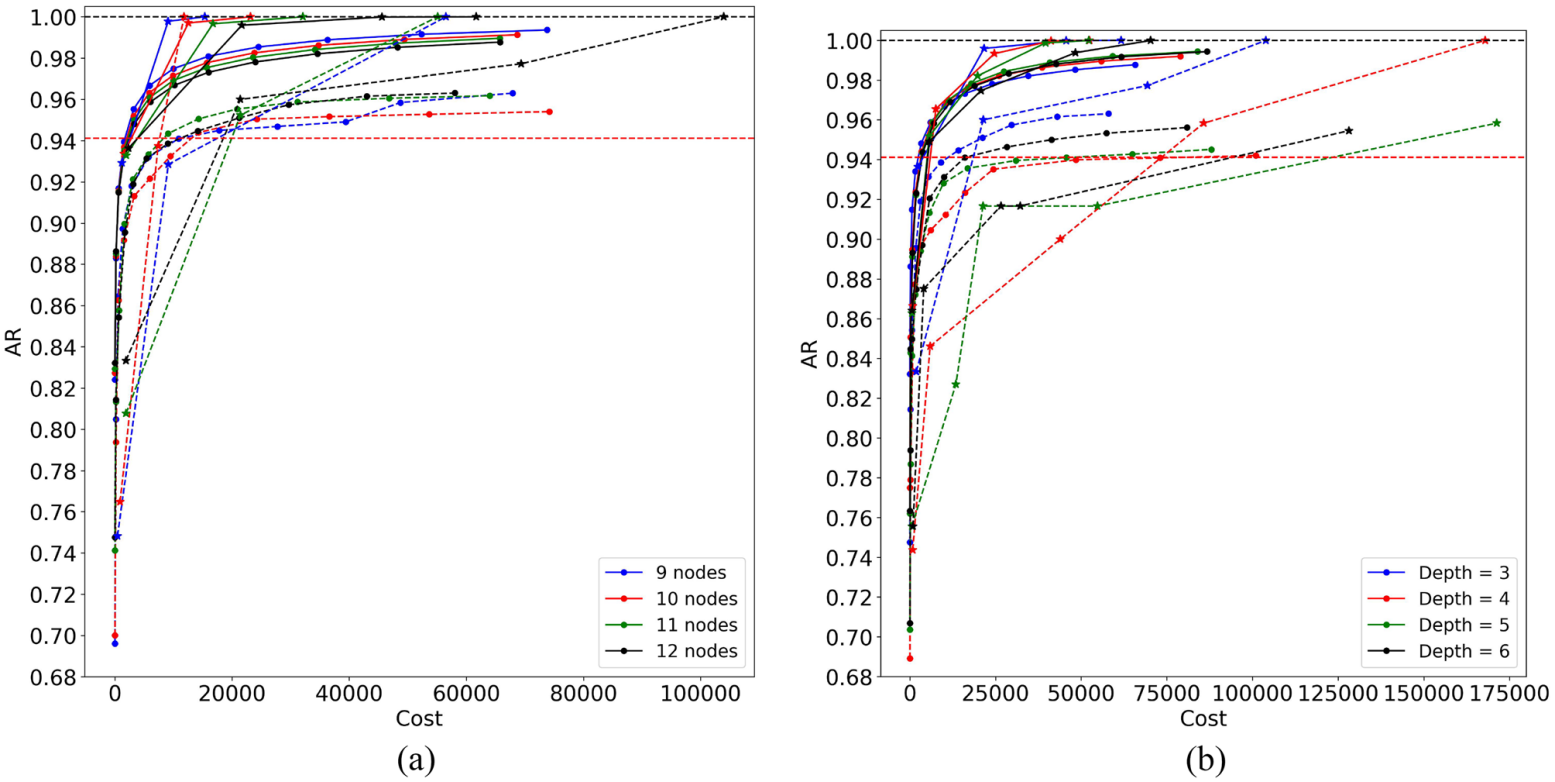
The data of Fig. 3, re-plotted using the cost defined in Eq. (9).

Higher dimensionality of the optimization space means more calls to QPU. Will MA-QAOA still be better than QAOA with this metric, i.e. will it have better AR for a given cost? In Fig. 4, we re-plotted the data of Fig. 3, using the metric of Eq. (9) as the argument. As one can see, overall, QAOA and MA-QAOA are closely matched in the region of smaller values of *p*. On average, QAOA achieves the desired accuracy level at a substantially smaller cost compared to MA-QAOA. The same is true for most (5 out of 7) data sets in the worst case. However, starting at the sufficiently high values of *p* ($p > 1$, data set dependent), MA-QAOA is able to achieve consistently larger ARs at any given cost. Considering the scaling with respect to the system size, the minimum value of *p* at which this happens is expected to grow.

## Conclusions

To summarize, the main novel contributions of this paper are the following

1. We investigated the numerical scaling of MA-QAOA for $p > 1$ and found that the advantage of MA-QAOA over QAOA is preserved even when multiple layers are considered, which allows it to converge to the desired accuracy in a much smaller number of layers, compared to QAOA. In addition to that, MA-QAOA was found to be less susceptible to performance decay associated with the problem size.
2. We found a new initialization strategy for MA-QAOA that allows to consistently outperform random initialization used in the previous studies. Additionally, this strategy guarantees that the performance of MA-QAOA will be no worse than that of QAOA.
3. We analyzed the prospects of MA-QAOA on a hypothetical fault-tolerant computer, where the depth of the circuit is no longer the primary concern, and found that in this scenario MA-QAOA no longer has an advantage over QAOA.

Additionally, we compared the performance of multiple parameter setting strategies for QAOA (Constant, TQA, Interp, Fourier, Greedy, Random) and MA-QAOA (Constant, Interp, QAOA Relax, Random QAOA, Random). Most of these initialization strategies have already been reported in the literature, but not all of them have been directly compared against each other on the same data sets. We consider this a minor, but interesting contribution.

These contributions are important because they establish the advantage of MA-QAOA over QAOA in a practically relevant setting of $p > 1$ and demonstrate that good angles can be found efficiently.

One limitation of the present study is that we considered random graphs only. It is possible that different classes of graphs might display different characteristics, therefore affecting the performance of MA-QAOA. Exploring this possibility could be a subject of future research.

As noted in this work, some QAOA angle initialization strategies do not work well for MA-QAOA. It is reasonable to assume that there may be MA-QAOA parameter initialization strategies that are not well-defined for QAOA due to the drastic difference in the number of parameters the algorithms require. As such, another direction for future work might include developing new angle initialization techniques for MA-QAOA that may not necessarily make sense for QAOA.

Another research direction could be to reduce the optimization cost of MA-QAOA by selecting only most relevant degrees of freedom.

### Supplementary Information

Supplementary Information.

## Data Availability

The full dataset of input graphs and raw results of calculations can be found at https://zenodo.org/records/12627513. Additionally, the source code can be found at https://github.com/GaidaiIgor/MA-QAOA.
