# Supplementary material for: Performance analysis of multi-angle QAOA for p > 1
Source: Sci Rep. 2024 Aug 14;14:18911. doi: 10.1038/s41598-024-69643-6 (PMC11324650; doi:10.1038/s41598-024-69643-6)
Supplement: Supplementary file 1 — Supplementary Information. [file 41598_2024_69643_MOESM1_ESM.pdf]

SUPPLEMENTAL INFORMATION

A. Comparison of QAOA Relax and Random initialization strategies

In the main text, we introduced a new initialization strategy for MA-QAOA, called QAOA Relax, and demonstrated its advantage over Random initialization strategy, used in the previous studies of MA-QAOA, but only on one data set. In Supplementary Figure 1, we show how this advantage changes as we increase the number of nodes or c-depth of the graphs. As one can see, the average AR difference steadily increases for all p as the number of nodes is increased, which makes this initialization strategy even more useful for larger graphs. The decrease in the difference of approximation ratios at larger values of p is due to the fact that QAOA Relax is already converged by $p \in \{2, 3\}$, as can be seen in Supplementary Figure 3, but Random converges later. The difference is even more pronounced when comparing the worst case ARs, achieving up to +0.14 AR on the considered data sets.

B. Comparison of different values for the Constant initialization strategy

In the main text we mentioned that we tried a few different values for the Constant initialization strategy. In Supplementary Figure 2 we show the detailed comparison between the ARs achieved with the each value on the data set with 9 nodes (same as in Supplementary Figure 1). The values from 0.05 to 0.4 have nearly the same AR on average, but can be distinguished in the worst case performance. The values of 0.1 and 0.2 have nearly the same worst-case performance too, but 0.2 converges somewhat faster, thus it was selected as the best value in the main text.

C. QAOA heuristics as a function of cost

It is interesting to repeat the analysis of section II A with the cost definition of Eq. 9 to take into account not only the number of layers, but also the number of QPU calls. The result of this is shown in Supplementary Figure 3. As one can see, the Constant initialization strategy still remains a winner, especially in the worst case, achieving not only the largest AR, but also using the smallest number of calls for the optimization procedure.

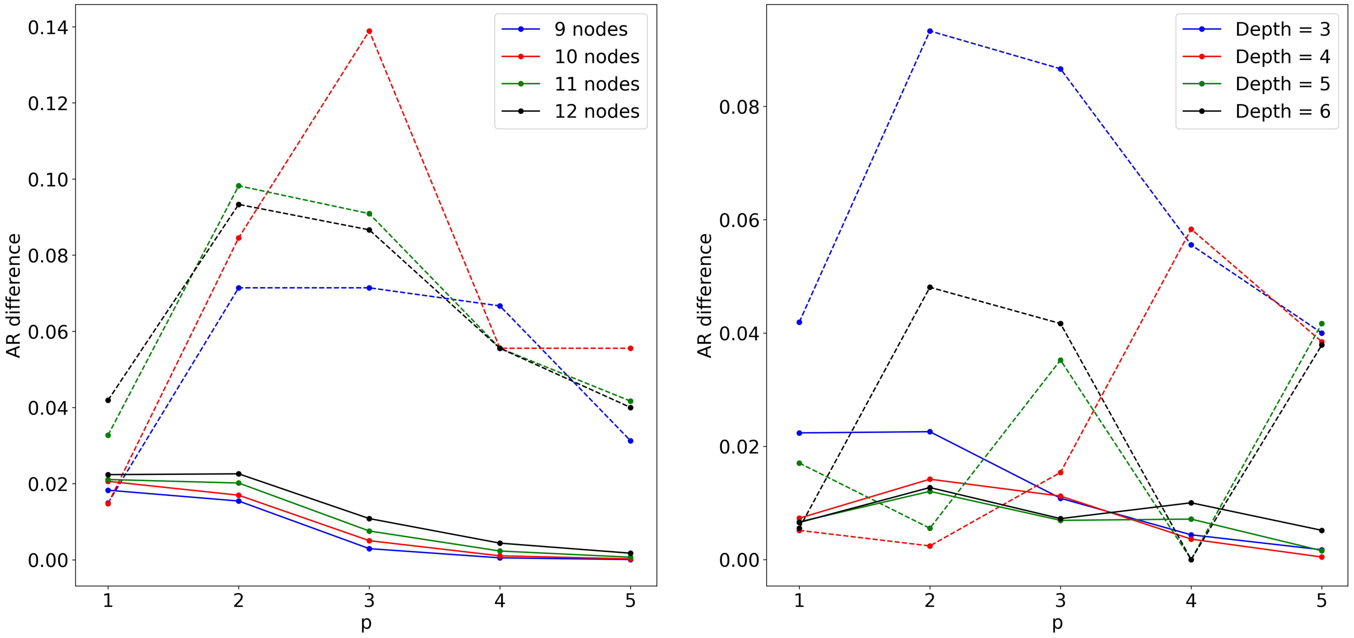

(a) Fixed c-depth = 3, varying number of nodes

(b) Fixed number of nodes = 12, varying c-depth

Supplementary Figure 1: AR of QAOA Relax relative to AR of Random. Solid (dashed) lines show differences in average (worst case) approximation ratio.

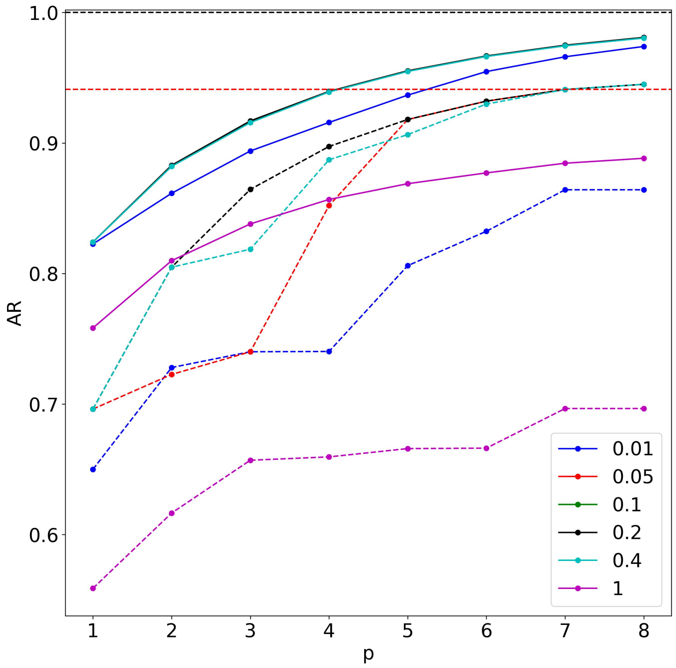

Supplementary Figure 2: AR obtained with different values of the Constant initialization strategy. Solid (dashed) lines show differences in average (worst case) approximation ratio.

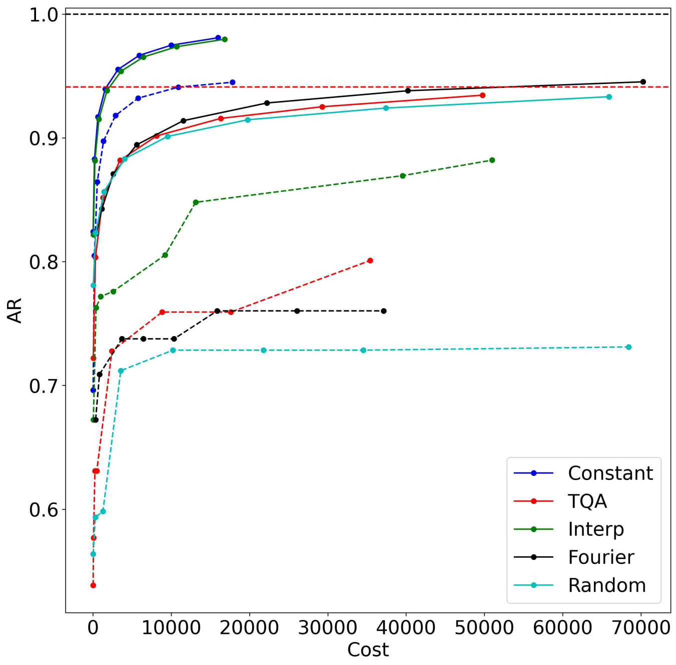

Supplementary Figure 3: AR vs cost for the QAOA initialization strategies considered in Figure 1a. Solid (dashed) lines show differences in average (worst case) approximation ratio.
